# Polymer-coated hexagonal upconverting nanoparticles: chemical stability and cytotoxicity

**DOI:** 10.3389/fchem.2023.1207984

**Published:** 2023-06-23

**Authors:** Vitalii Patsula, Dana Mareková, Pavla Jendelová, Mykhailo Nahorniak, Oleksandr Shapoval, Petr Matouš, Viktoriia Oleksa, Rafał Konefał, Magda Vosmanská, Lucia Machová-Urdziková, Daniel Horák

**Affiliations:** ^1^ Institute of Macromolecular Chemistry, Czech Academy of Sciences, Prague, Czechia; ^2^ Institute of Experimental Medicine, Czech Academy of Sciences, Prague, Czechia; ^3^ Department of Neurosciences, Second Faculty of Medicine, Charles University, Prague, Czechia; ^4^ Center for Advanced Preclinical Imaging, First Faculty of Medicine, Charles University, Prague, Czechia; ^5^ Department of Analytical Chemistry, University of Chemistry and Technology Prague, Prague, Czechia

**Keywords:** luminescence, upconversion, nanoparticles, lanthanides, dissolution, cell viability, uptake

## Abstract

Large (120 nm) hexagonal NaYF_4_:Yb, Er nanoparticles (UCNPs) were synthesized by high-temperature coprecipitation method and coated with poly(ethylene glycol)-alendronate (PEG-Ale), poly (*N*,*N*-dimethylacrylamide-*co*-2-aminoethylacrylamide)-alendronate (PDMA-Ale) or poly(methyl vinyl ether-*co*-maleic acid) (PMVEMA). The colloidal stability of polymer-coated UCNPs in water, PBS and DMEM medium was investigated by dynamic light scattering; UCNP@PMVEMA particles showed the best stability in PBS. Dissolution of the particles in water, PBS, DMEM and artificial lysosomal fluid (ALF) determined by potentiometric measurements showed that all particles were relatively chemically stable in DMEM. The UCNP@Ale-PEG and UCNP@Ale-PDMA particles were the least soluble in water and ALF, while the UCNP@PMVEMA particles were the most chemically stable in PBS. Green fluorescence of FITC-Ale-modified UCNPs was observed inside the cells, demonstrating successful internalization of particles into cells. The highest uptake was observed for neat UCNPs, followed by UCNP@Ale-PDMA and UCNP@PMVEMA. Viability of C6 cells and rat mesenchymal stem cells (rMSCs) growing in the presence of UCNPs was monitored by Alamar Blue assay. Culturing with UCNPs for 24 h did not affect cell viability. Prolonged incubation with particles for 72 h reduced cell viability to 40%–85% depending on the type of coating and nanoparticle concentration. The greatest decrease in cell viability was observed in cells cultured with neat UCNPs and UCNP@PMVEMA particles. Thanks to high upconversion luminescence, high cellular uptake and low toxicity, PDMA-coated hexagonal UCNPs may find future applications in cancer therapy.

## 1 Introduction

Upconverting nanoparticles (UCNPs) have attracted much attention already since 1966, when the general principles of photon upconversion were first discovered (Auzel, 2004). Currently, luminescent lanthanide-doped upconverting nanoparticles (UCNPs) are widely used in medicine for biological imaging and diagnosis of serious diseases (Li et al., 2020). UCNPs have the advantage that they can be excited by relatively long wavelengths, namely, near-infrared (NIR) light, typically in the 700–1,000 nm spectral range, which penetrates deep into tissues and converts low-energy NIR photons into high-energy UV-visible light (Hamblin, 2018; Wen et al., 2018). UCNPs typically consist of NaYF_4_ as a host matrix, which is usually co-doped with a sensitizer (Yb^3+^ or Nd^3+^) and an activator (Er^3+^, Tm^3+^ or Ho^3+^) (Ye et al., 2015). NaYF_4_ can exist under normal pressure in two polymorphic phases, cubic and hexagonal, the latter showing an order of magnitude higher upconversion efficiency than the former (Park et al., 2013). The crystallinity of UCNPs, and thus the lattice structure, is also affected by size, resulting in a change in luminescent properties (Sun et al., 2014). A strong dependence on the size of upconversion NaYF_4_:Yb^3+^,Er^3+^ nanocrystals was observed; the larger the particles, the higher the emission (Schietinger et al., 2009), which also affects the synthesis strategy of UCNPs. Methods developed for the preparation of hexagonal-phase UCNPs with uniform size (Wang et al., 2012), which is advantageous for biomedical applications, include thermal decomposition (Xie et al., 2013), hydrothermal/solvothermal synthesis with phase transfer (Wang et al., 2009), microemulsion (Shan et al., 2011), sol-gel processing (Yao et al., 2016) and microwave synthesis (Egatz-Gomez et al., 2022).

Despite the many undeniable advantages of UCNPs, such as large anti-Stokes shift, narrow emission bandwidth, no photobleaching, no blinking, deep detection ability, and low damage to biological tissues, these particles also have some pitfalls. For example, they have relatively low quantum yields that need to be increased for future applications in PDT, bioimaging, sensing, theranostics and optogenetics (Jones et al., 2021). In contrast to bulk materials, higher photoluminescence efficiency of UCNPs can be achieved, for example, by better selection of the host lattice (Fischer et al., 2014), combination and appropriate concentration of rare earth dopants (Kaiser et al., 2017), improvement of nanoparticle crystallinity (Zhou et al., 2015), or introduction of core-shell structures (Fischer et al., 2016) to suppress the surface quenching characteristic of lanthanide nanoparticles (Wang et al., 2010); plasmonic and photonic structures (Park et al., 2015) and dye sensitizers can also be used (Wisser et al., 2018). In addition, UCNPs used in a living organism must be biocompatible, hydrophilic, dispersible in biological media, poorly soluble and, last but not least, chemically and colloidally stable (Liang et al., 2020). Initially, UCNPs were thought to be chemically inert, but recently an increasing number of studies have demonstrated their dissolution in aqueous media at high dilution (Abdul Jalil and Zhang, 2008), leading to the release of F^−^ and Ln^3+^ ions (Barbier et al., 2010; Lisjak et al., 2016; Plohl et al., 2017). This highlights the need for more detailed studies addressing the chemical stability and toxicity of UCNPs (Oliveira et al., 2019). In particular, the effect of particles size on their dissolution is only reported for UCNPs <50 nm in diameter and data on the degradation of larger UCNPs are lacking. The dissolution of large UCNPs (>100 nm) can be expected to be lower compared to small particles (<50 nm), which are characterized by a larger surface-to-volume ratio than large particles. As a result, the upconversion fluorescence intensity of the latter particles can be maintained for longer periods of time, which significantly affects their use in biomedical applications. To the best of our knowledge, so far, only a minimum of works has dealt with monodisperse UCNPs of 100–200 nm in size, except for hollow microtubes, rods and peanut-shaped particles. It can be assumed that they will dissolve mainly in the cell and their dissolution will then depend on their cellular uptake and on the surface-to-volume ratio, which will be different for various particle morphologies. In view of the above facts, subsequent surface protection of UCNPs against aggregation, precipitation or dissolution is necessary, consisting of their treatment with hydrophilic polymers, chelating agents, bioconjugations, hybrid materials, etc. (Boyer et al., 2006; Li et al., 2015). Examples of polymers that provide colloidal stability of UCNPs are poly (ethylene glycol) (PEG) (Pichaandi et al., 2011), silica (Johnson et al., 2010), polyvinylpyrrolidone (MacKenzie et al., 2022), poly (acrylic acid) (Jin et al., 2011), polyethyleneimine (Shao et al., 2018), etc. The conjugation of specific biomolecules such as antibodies (Ramírez-García et al., 2018), nucleic acids (Mao et al., 2017) or proteins (Kamimura et al., 2008) required for a given bioanalytical application is facilitated by functional, often hydroxyl, carboxyl or amino groups of the polymers (Mi et al., 2010; Wilhelm et al., 2015). The surface coating influences the physicochemical properties of nanoparticles, especially their chemical and colloidal stability, interactions with biological systems, and ultimately affects their cellular uptake and toxicity. The coating can reduce cell viability by facilitating cellular uptake by changing the ξ-potential or improving colloidal stability; in contrast, if uncoated particles aggregate, they are less likely to enter cells. If surface coatings are not firmly bound to particles, they may be released into body fluids during application, increasing toxicity.

The aim of this work was to prepare highly sensitive multicolor fluorescent nanoparticles combining upconversion luminescence and fluorescence imaging using fluorescein-5-isothiocyanate (FITC) to follow particle uptake during cell culture. The combination of two luminescent agents with excitation and emission in the visible to NIR region makes these particles an ideal contrast for *in vitro* and *in vivo* visualization of various biological processes. Multicolor particles were prepared by attaching FITC to neat and poly(ethylene glycol)-alendronate (PEG-Ale)-, poly (*N*,*N*-dimethylacrylamide-*co*-2-aminoethylacrylamide)-alendronate (PDMA-Ale)- and/or poly (methyl vinyl ether-*co*-maleic acid) (PMVEMA)-modified hexagonal-phase NaYF_4_:Yb^3+^,Er^3+^ UCNPs that were larger than 100 nm in size. The stability of these nanomaterials was studied under various conditions in aqueous media like water, 0.01 M phosphate buffered saline (PBS; pH 7.4), Dulbecco’s modified Eagle’s medium (DMEM) or artificial lysosomal fluid (ALF). The concentrations of released F ¯ and Y^3+^ ions were determined by combined fluoride electrode, UV-Vis spectroscopy and inductively coupled plasma mass spectrometry (ICP-MS). Internalization of UCNPs into rMSCs or C6 cells was monitored using a multiphoton laser scanning microscope, epifluorescent microscopy and cytotoxicity was tested using the Alamar Blue.

## 2 Experimental

### 2.1 Materials

Octadec-1-ene (OD; 90%), NH_4_F (99.99%), anhydrous YCl_3_ and YbCl_3_ (99%), ErCl_3_∙6 H_2_O (99%), *N,N*-dimethylacrylamide (DMA; 99%), fluorescein isothiocyanate (FITC), and phosphate buffered saline (PBS) were purchased from Sigma-Aldrich (St Louis, MO, United States). Sodium trihydrate of (4-amino-1-hydroxy-1-phosphonobutyl)phosphonic acid (alendronate; Ale) was obtained from TCI (Tokyo, Japan). Poly (methyl vinyl ether-*co*-maleic acid) (PMVEMA; *M*_w_ = 60,000 g/mol) was obtained from Scientific Polymer Products (Ontario, NY, United States). Oleic acid (OA; 98%), methanol (99.5%), hexane (99.5%), dichloromethane (99.9%), and paraformaldehyde were from Lach-Ner (Neratovice, Czech Republic). Ale-modified PEG (PEG-Ale) and poly (*N*,*N*-dimethylacrylamide-*co*-2-aminoethylacrylamide)-alendronate (PDMA-Ale) were prepared as described in literature (Kostiv et al., 2017; Oleksa et al., 2020; Nahorniak et al., 2023). Dulbecco’s modified Eagle’s medium (DMEM), Alamar Blue assay and 4′,6-diamidino-2-phenylindole (DAPI) were purchased from Thermo Fischer Scientific (Waltham, MA, United States). Fetal bovine serum (FBS) was from Merck (Darmstadt, Germany) and primocin and penicillin streptomycin were from Gibco, Life Technologies (Grand Island, NY, United States). Artificial lysosomal fluid (ALF, pH 4.5) was prepared as previously reported (Colombo et al., 2008). Suprapur^®^ nitric acid was purchased from Merck (Kenilworth, NJ, United States). Mesenchymal stromal cells (rMSCs) were isolated from rat bone marrow according to the previous report (Nahorniak et al., 2023). C6 cells were kindly provided by Dr. Čestmír Altaner from the Cancer Research Institute SAS (Bratislava, Slovakia). All other reagent grade chemicals were purchased from Sigma-Aldrich and used as received. Polyethersufone filter (MWCO 30 kg/mol) was obtained from VWR (Radnor, PA, United States). Ultrapure water (conductivity ˂0.1 μS/cm) was obtained on a Milli-Q Gradient A10 purification system (Millipore; Molsheim, France) and used throughout the experimental work.

### 2.2 Synthesis of hexagonal NaYF_4_:Yb^3+^,Er^3+^ nanoparticles (UCNPs)

UCNPs with hexagonal-phase were prepared by high-temperature coprecipitation method. To a solution of OA (6 mL) in OD (15 mL), YCl_3_ (0.78 mmol), YbCl_3_, (0.2 mmol) and ErCl_3_∙6 H_2_O (0.02 mmol) were added under magnetic stirring. The reaction mixture was heated to 160°C under vacuum (2.1 kPa) and maintained at this temperature for 30 min. The solution was then cooled to room temperature (RT) under Ar atmosphere and a solution of NH_4_F (148 mg; 4 mmol) and NaOH (100 mg; 2.5 mmol) in methanol (8 mL) was added. To evaporate the methanol, the reactor pressure was slowly reduced to 2.1 kPa while stirring and the mixture was heated to 65°C. The reaction temperature was then slowly raised to 120°C and maintained at this value for 30 min to evaporate the residual water. Finally, the mixture was heated at 300°C for 1.5 h under Ar atmosphere and cooled to RT. The UCNPs were separated by centrifugation (3,460 rcf) for 1 h, washed twice with a hexane/ethanol (1:1 v/v) mixture, three times with ethanol, twice with ethanol/water (1:1 v/v), and twelve times with water (14 mL each), and redispersed in water.

### 2.3 Surface modification of hexagonal UCNPs with PEG-Ale, P(DMA-AEA)-Ale, and PMVEMA

Modification of UCNPs with polymers was performed according to a previously published procedure (Nahorniak et al., 2023). Briefly, a dispersion of UCNPs (30 mg) in water (1.64 mL) was added dropwise to an aqueous solution (2 mL) of PEG-Ale (*M*_w_ = 5,000 g/mol) or PDMA-Ale (15 mg; *M*_w_ = 11,000 g/mol) under sonication (Ultrasonic Homogenizer UP200S Hielscher; 20% power) for 1 min. The mixture was magnetically stirred at RT for 24 h. The resulting polymer-coated UCNPs denoted as UCNP@Ale-PEG and UCNP@Ale-PDMA were sedimented by centrifugation and washed twice with water (1.5 mL) followed by centrifugation (14,100 rcf).

UCNP@PMVEMA nanoparticles were prepared by adding aqueous dispersion of UCNPs (15 mg; 1 mL) to aqueous PMVEMA solution (50 mg/mL; 15 mL; pH 7.4 adjusted by adding 2 M NaOH) with shaking at RT for 30 min and further stirring at 70°C for 16 h. Nanoparticles were sedimented by centrifugation (14,100 rcf) and washed with water three times.

### 2.4 Synthesis of FITC-alendronate and modification of hexagonal UCNPs

For the preparation of FITC-alendronate (FITC-Ale), FITC (105 mg) was dissolved in dimethyl sulfoxide (1 mL), a solution of Ale (70 mg) in 0.2 M sodium carbonate-bicarbonate buffer (3 mL; pH 10.4) was added, and the mixture was stirred at RT for 72 h in the dark. Then, the resulting FITC-alendronate (FITC-Ale) was purified by gel filtration on a Sephadex G-25 column with water as eluent and the solution was passed through a column filled with sulfonated polystyrene to replace Na^+^ with H^+^ ions in the phosphonate groups of FITC-Ale. Finally, water was removed at 40°C on a vacuum rotary evaporator. The structure of FITC-Ale was confirmed by ^1^H NMR (SI Supporting Information, Supplementary Figure S1); the purity was 52% and the yield was 29 mg.

In the next step, neat UCNPs or UCNPs coated with PEG-Ale and PDMA-Ale were functionalized with FITC-Ale. An aqueous solution of FITC-Ale (0.05 mg; 0.5 mL) was added to an aqueous dispersion (3.64 mL) of particles (30 mg) and the mixture was stirred at RT for 21 h in the dark. In the case of UCNP@PMVEMA particles, an aqueous solution of FITC-Ale (0.5 mg; 0.225 mL) was added 30 min after the addition of the PMVEMA solution to the neat UCNPs under the same conditions as described above. The resulting FITC-modified particles were separated by centrifugation and washed twice with water (1.5 mL) followed by centrifugation (14,100 rcf).

### 2.5 Dissolution of hexagonal UCNPs

Dispersion of UCNPs in water, 0.01 M PBS (pH 7.4), DMEM or ALF (1 mg/mL) was placed in a plastic vial sealed with a rubber septum and allowed to age at 25 or 37°C for a period of time with agitation (250 rpm). Subsequently, the dispersion was centrifuged (14,129 rcf) for 30 min to separate UCNPs and the supernatant was filtered (MWCO 30 kg/mol) to remove residual particles. A combined fluoride electrode (Thermo Fisher Scientific; Waltham, MA, United States) was used to measure the dissolution of UCNPs expressed as the concentration of released F ¯ ions in the medium according to the manufacturer’s instructions. The molar fraction of F ¯ ions was expressed as the mean ± standard error of the mean (*n* = 3).

The concentration of Y^3+^, Yb^3+^ and Er^3+^ ions leached from neat and polymer-coated UCNPs was determined spectrophotometrically according to our previous publication (Nahorniak et al., 2023). Briefly, supernatants from particle dispersions were separated via centrifugation (14,010 rcf) for 30 min and mixed with xylenol orange buffer solution (2 mL; pH 5.8). The concentration of free Y^3+^, Yb^3+^ and Er^3+^ released from the UCNPs was measured by UV-Vis spectroscopy at 350–650 nm from the ratio of absorbance at 570 and 443 nm. The calibration curve was obtained as the dependence of the integrated intensity ratios of absorption bands from 18 μM xylenol orange in acetate buffer (pH 5.8) at different concentrations of YCl_3_ (0–70 μM Y^3+^).

### 2.6 Inductively coupled plasma mass spectrometry (ICP-MS)

ICP-MS measurements were performed on a PerkinElmer NexION 350D ICP-MS instrument (Woodbridge, Canada) equipped with Universal Cell Technology™. The sample system consisted of an internal peristaltic pump with Tygon^®^ tubing (0.38 mm inner diameter), a polytetrafluoroethylene concentric nebulizer and a 100-mL glass cyclone spray chamber.

After 24 h incubation with all tested UCNPs at a concentration of 20 μg/mL, rMSCs or C6 cells were treated with concentrated nitric acid (3 mL) and the mixture was placed in a Teflon digestion vessel (Speedwave 4; Berghof, Germany). After dilution, digested sample was spiked with internal standard solution (^100^Rh) and placed in a 50-mL volumetric flask. Calibration solution and internal standard solution were prepared from a starting concentration of 1.000 ± 0.002 g/L (Merck, Darmstadt, Germany).

### 2.7 Determination of cytotoxicity

The cell cultures were prepared as previously reported (Nahorniak et al., 2023) and the cytotoxicity of all prepared UCNPs was determined by Alamar Blue assay. Briefly, rMSCs and C6 cell line were incubated with UCNPs for 24 h in complete culture medium, after which the medium was aspirated and a 10% Alamar Blue solution in culture medium was added and incubation continued for 3 h. The solution was pipetted into 96-well plates and fluorescence was measured using a FLUOstar Omega reader (BMG LABTECH; Ortenberg, Germany) at an excitation of 530 nm and emission of 590 nm. In the meantime, fresh culture medium was added to the cells and left for another 48 h; fluorescence was measured after 72 h as a percentage of control (readouts of nanoparticle-free cells).

### 2.8 Microscopy imaging

Cells grown on coverslips were incubated with UCNPs (20 μg/mL) for 24 h, fixed with 4% paraformaldehyde for 20 min, stained with DAPI and mounted. The presence of UCNPs in rMSCs or C6 cells was monitored using an Olympus FV1200 MPE multiphoton laser scanning microscope (Tokyo, Japan). Briefly, cells were fixed with 4% paraformaldehyde in PBS, stained with DAPI and light-field images were taken at 980 nm excitation and 540 nm emission using an infrared pulsed laser with negative chirp for multiphoton excitation. DAPI-stained cells were observed in the blue channel using a 405 nm laser diode and the bright field and the blue channel were superimposed. FITC-modified UCNPs were imaged using a Zeiss fluorescence microscope (Oberkochen, Germany) in a green filter (488 nm).

### 2.9 Equipment

Micrographs of the synthesized UCNPs were recorded with a Tecnai Spirit G2 transmission electron microscopy (TEM; FEI; Brno, Czech Republic). The TEM was coupled with energy-dispersive X-ray (EDX) detector (Mahwah, NJ, United States). Particles were also characterized using a Thermo Nicolet 870 FTIR spectrometer (Madison, WI, United States) equipped with a liquid nitrogen-cooled mercury cadmium telluride detector using a GoldenGate single reflection diamond ATR system (Specac; Orpington, United Kingdom). Hydrodynamic size, polydispersity (*PD*) and ξ-potential were measured using dynamic light scattering (DLS; ZSU 5700 Zetasizer Ultra Instrument; Malvern Instruments; Malvern, United Kingdom). ^1^H NMR spectra were measured on a Bruker Avance III 600 spectrometer (Billerica, MA, United States). Thermogravimetric analysis (TGA) was conducted on a Perkin Elmer TGA 7 analyzer (Norwalk, CT, United States); the particles were heated in air from RT to 650°C at 10°C/min. UV-Vis spectra were recorded on a Specord 250 Plus UV-Vis spectrophotometer (Analytik; Jena, Germany). Emission and excitation spectra were recorded using a FS5 Edinburgh Instruments spectrofluorometer (Livingston, United Kingdom) coupled with UV-Vis Xe lamp and 980 nm CW laser with 2 W output power (MDL-III-980).

## 3 Results and discussion

### 3.1 Polymer-coated hexagonal UCNPs

**FIGURE 1:**
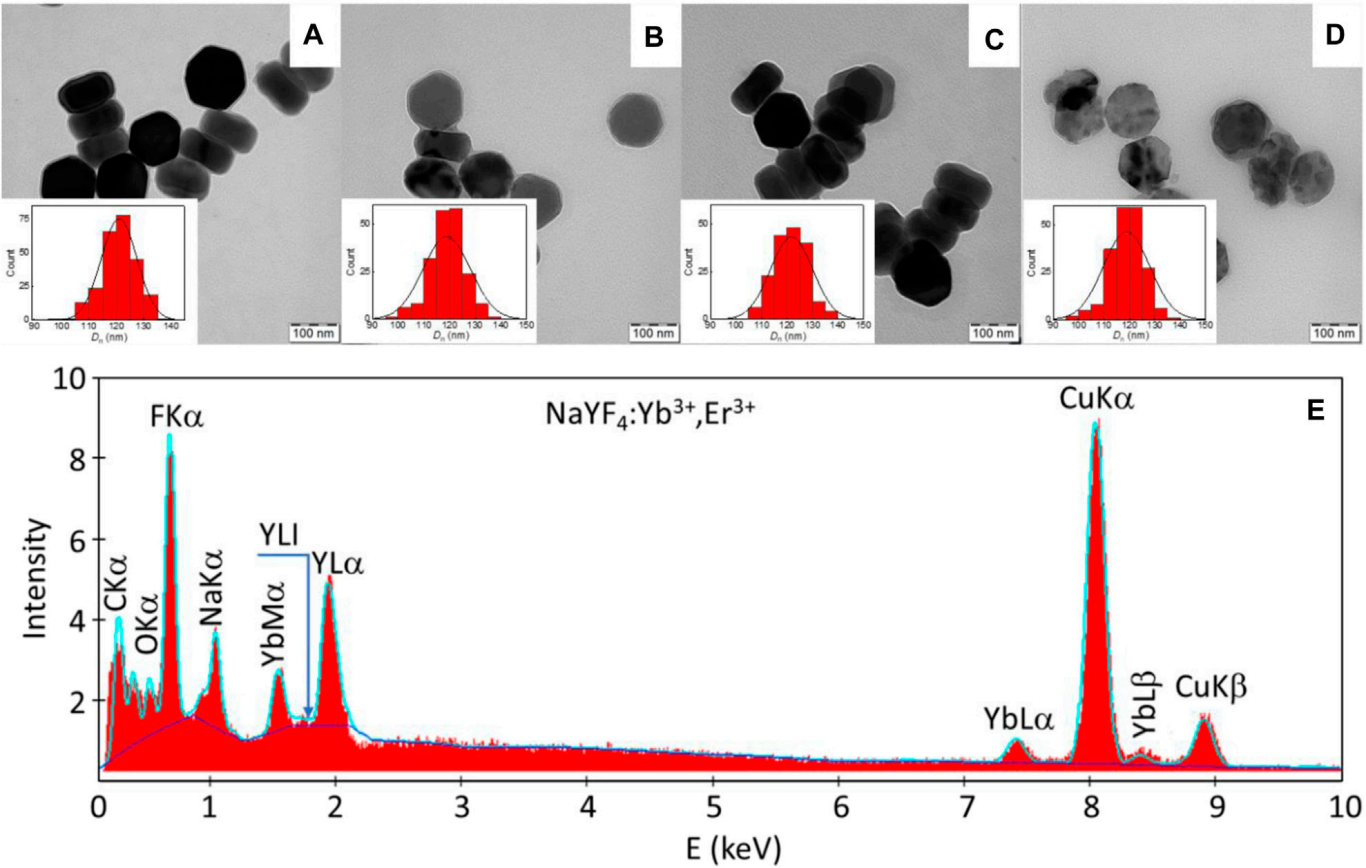
**(A)** TEM micrographs of **(A)** UCNPs, **(B)** UCNP@Ale-PEG, **(C)** UCNP@Ale-PDMA, **(D)** UCNP@PMVEMA and **(E)** TEM/EDX analysis of hexagonal UCNPs.

The UCNPs were synthetized by a high temperature coprecipitation method with rigorous removal of volatile solvents, which resulted in the formation of fewer nuclei than in the preparation of small (25 nm) spherical particles (Nahorniak et al., 2023). The TEM image showed that the UCNPs consisted of larger particles transformed into hexagonal plates that had the uniform size with dispersity *Ð* ∼1 (Figure 1A; Table 1). The average diameter of the plates was ∼120 nm and their thickness was 68 nm. The hydrodynamic size was 174 nm and the ξ-potential was positive (28 mV; Table 1, Supplementary Figure S2). The presence of Y, Yb and F in the particles was confirmed by TEM/EDX spectra, which showed large peaks at ∼0.68, 1.04 and 1.91 keV corresponding to F, Na and Y atoms, respectively (Figure 1E). Peaks at 0.23 and 8.04 keV were attributed to C and Cu atoms from the supporting TEM grid. The smaller peaks at 1.54, 7.4 and 8.42 keV belonged to Yb atoms, while the signal of Er atoms (∼6.9 keV) was not visible due to their low concentration.

**TABLE 1:** Characterization of differently coated UCNPs and their dissolution rates in PBS.

Particles	*D*_n_ (nm)	*Đ*	*D*_h_ (nm)	*PD*	ζ-potential (mV)	Coating^a^ (wt%)	Dissolution rate (mol%/h)	
							25°C	37°C
UCNPs	121	1.01	174 ± 1	0.11	28 ± 3	0.2	0.54 ± 0.06	1.3 ± 0.3
UCNP@Ale-PEG	119	1.02	158 ± 3	0.09	4 ± 1	1.5	0.32 ± 0.02	0.8 ± 0.2
UCNP@Ale-PDMA	122	1.01	160 ± 3	0.07	22 ± 2	3.3	0.31 ± 0.01	1.0 ± 0.2
UCNP@PMVEMA	119	1.01	234 ± 7	0.10	−46 ± 6	14.4	0.08 ± 0.01	0.5 ± 0.1

UCNPs, upconverting NaYF_4_:Yb^3+^, Er^3+^. Nanoparticles; PEG-Ale, poly(ethylene glycol)-alendronate; PDMA-Ale, poly(N,N-dimethylacrylamide)-alendronate; PMVEMA, poly (methyl vinyl ether-*co*-maleic acid); *D*_n_, number-average diameter (TEM); *Ð*, dispersity *D*_w_/*D*_n_ (TEM); *D*_h_, hydrodynamic diameter (DLS); *PD*, polydispersity (DLS).

^a^ According to TGA.

**FIGURE 2:**
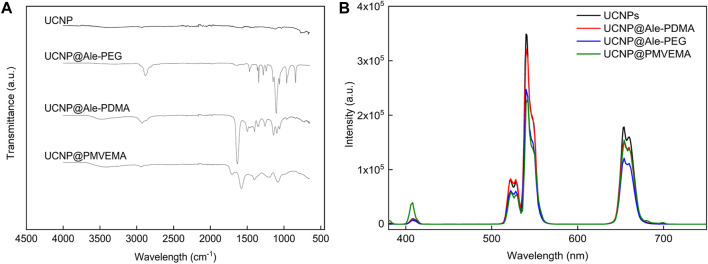
**(A)** ATR FTIR spectra and **(B)** upconversion photoluminescence emission spectra of uncoated and polymer-coated hexagonal UCNPs. The upconversion spectra were measured at concentration of 4 mg of particles/mL of water, excitation at 980 nm and laser power density of 0.25 W/cm^2^.

To ensure the chemical and colloidal stability of hexagonal UCNPs in aqueous media used in biological experiments, the particles were protected against dissolution by coating with polymers, namely, PEG-Ale, PDMA-Ale and PMVEMA; the hydrodynamic particle size then reached ∼160–230 nm and the polydispersity *PD* was ∼0.1, characterizing a narrow particle size distribution (Figures 1B-D, Table 1, Supplementary Figure S2A). Compared to UCNP@PMVEMA particles, the correlation functions of UCNP@Ale-PEG and UCNP@Ale-PDMA decayed faster confirming their smaller size (Supplementary Figure S2B). The ξ-potential of UCNP@Ale-PEG, UCNP@Ale-PDMA and UCNP@PMVEMA particles was 4, 22 and −46 mV, respectively, (Table 1, Supplementary Figure S2C). This was due the electroneutrality of PEG and the positive and negative charges of PDMA and PMVEMA, respectively. At the same time, negatively charged particles are known to have a lower frequency of binding to plasma proteins, i.e., they avoid clearance by the reticuloendothelial system. According to TGA, the amount of PEG, PDMA and PMVEMA coating was ∼2, 3 and 14 wt% (Table 1). It should still be noted that PDMA contained a reactive *N*-(2-aminoethyl)acrylamide comonomer suitable for potential attachment of biomolecules. The ATR FTIR spectra of hexagonal UCNPs, UCNP@Ale-PEG, UCNP@Ale-PDMA and UCNP@PMVEMA particles confirmed the presence of polymers on the nanoparticle surface, where the characteristic ν(OH), ν(C-H) symmetric and asymmetric, ν(CH_2_) symmetric and C-O stretching vibrations were observed at ∼3,450–3,300, 2,927–2,884, 2,850–2,860 and 1,105–1,080 cm^-1^, respectively (Figure 2A). The spectrum of hexagonal UCNPs@Ale-PDMA exhibited absorption band at 1,633 cm^-1^, which was attributed to ν(C=O) stretching vibrations of the amide DMA (Oleksa et al., 2020). Then, after modification of the hexagonal UCNPs by PMVEMA, the asymmetric (1,578 cm^-1^) and symmetric (1,403 cm^-1^) stretching vibrations of the COOH groups of PMVEMA were found in the spectrum (Ahmad et al., 2020).

### 3.2 Luminescence of UCNPs

The upconversion luminescence of the neat and polymer-coated hexagonal UCNPs in water was determined under excitation in the near-infrared region at a wavelength of 980 nm (Figure 2B). The characteristic upconversion emission of Er^3+^ was observed, which was attributed to the transitions ^2^H_11/2_ → ^2^I_15/2_ (409 nm), ^4^S_3/2_ → ^2^I_15/2_ (525 nm), ^2^H_9/2_ → ^4^I_15/2_ (542 nm), and ^4^F_9/2_ → ^2^I_15/2_ (656 nm). The upconversion emission spectra of the hexagonal nanoparticles (120 nm in diameter) showed similar peaks and emission intensities to those observed for previously described spherical 25-nm particles (Supplementary Figure S3) (Nahorniak et al., 2023). However, the uncoated large hexagonal UCNPs exhibited ∼30 times higher emission intensity at 542 nm than the small spherical UCNPs, making them more attractive and useful for bioassays and biological imaging. Uncoated and PEG-Ale- and PDMA-Ale-coated UCNPs exhibited weaker upconversion emission intensity than UCNP@PMVEMA, independent of the thickness of the PDMA and PEG coating. This demonstrated the quenching effect caused by irregularities in the coating of these particle that allowed direct access of water to the particle surface. The UCNP@PMVEMA particles had the highest upconversion emission intensity due to their largest hydrodynamic diameter of all particles.

### 3.3 Colloidal stability of UCNPs

The colloidal stability of neat and polymer-modified UCNPs in water, PBS and DMEM was monitored by analyzing their *D*_h_ and ζ-potential as a function of time. No significant changes in *D*_h_ and ζ-potential were observed for any of the polymer-coated nanoparticles incubated in water at 25°C and 37°C for 168 h, demonstrating their good colloidal stability (Supplementary Figures S4A, B and Supplementary Figures S5A, B). Similar results were observed for neat UCNPs, but after 168 h at 37°C the particles started to aggregate and their hydrodynamic diameter increased from 120 to 220 nm. This was attributed to a decrease in their ζ-potential, as these nanoparticles were only stabilized by electrostatic repulsions (Supplementary Figure S5A). In PBS, the *D*_h_ of UCNP@Ale-PEG increased after 24 and 6 h of incubation at 25°C and 37°C, respectively. UCNP@Ale-PDMA particles started to aggregate immediately after contact with PBS, and their *D*_h_ was >1,000 nm. In both cases, the aggregation was probably due to the small amount and rapid exchange of polymer coatings with phosphate ions, as previously observed for PEG-phosphate-modified UCNPs (Boyer et al., 2010). The ζ-potential of both UCNP@Ale-PEG and UCNP@Ale-PDMA approached zero due to the formation of the counterion layer (Supplementary Figure S5C, D). UCNP@PMVEMA particles showed very good colloidal stability in PBS at both temperatures for 168 h, as there were no noticeable changes in their *D*_h_ and ζ-potential. Also, neat UCNPs, UCNP@Ale-PEG and UCNP@PMVEMA particles were colloidally stable in DMEM at 37°C and their *D*_h_ and *PD* were constant for 168 h (Supplementary Figure S6A, B). In contrast, UCNP@Ale-PDMA particles were unstable in DMEM and their *D*_h_ and *PD* increased due to opsonization with proteins and aggregation.

### 3.4 Dissolution of UCNPs

**FIGURE 3:**
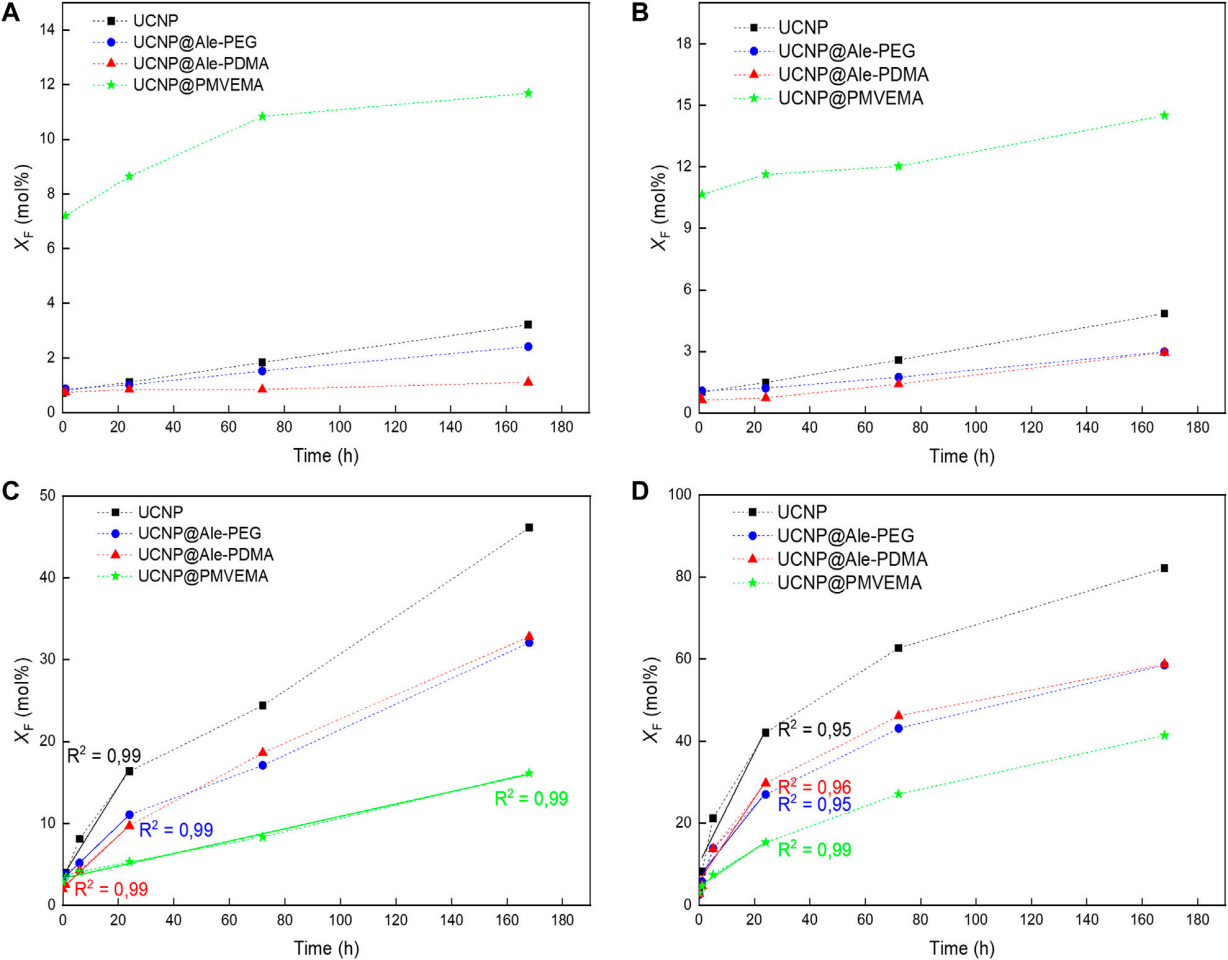
Time dependences of F ¯ ion molar fraction (*X*_F_) in supernatants. Hexagonal UCNPs were stored in **(A, B)** water and **(C, D)** PBS (pH 7.4) at **(A, C)** 25 and **(B, D)** 37°C. The standard errors of the means of *X*_F_ in water ranged from 0.02 to 0.026 and in PBS from 0.02 to 0.24 mol%.

**FIGURE 4:**
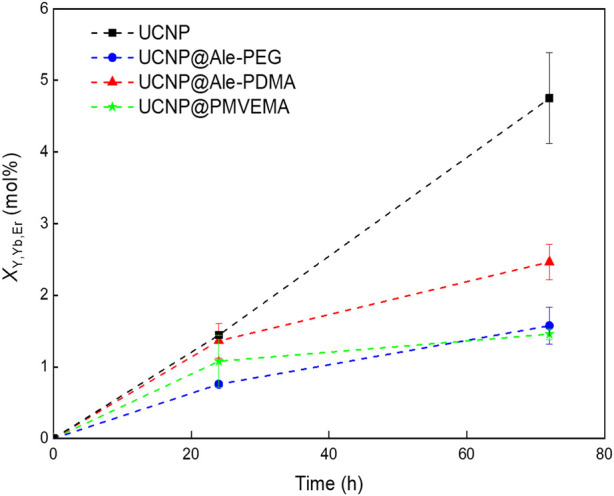
Time dependence of Y^3+^, Yb^3+^ and Er^3+^ ion molar fraction (*X*_Y,Yb,Er_) in supernatants. Hexagonal UCNPs (1 mg/mL) were aged in PBS at 37°C.

The dissolution of neat and polymer-coated hexagonal UCNPs in water and PBS at 25°C and 37°C was measured with a fluoride ion selective electrode from time-depended changes in the concentration of F ¯ ions in the supernatants. The F ¯ ion leakage was determined as the molar fraction of dissolved F ¯ ions (*X*_F_) relative to the total amount of fluorine in the nanoparticles with a nominal composition of NaF_4_:Y_0.78_:Yb_0.20_,Er_0.02_ (Figures 3A–D). It can be seen that at higher temperatures, *X*_F_ increased (Figures 3B, D) due to the better solubility of NaYF_4_ (Lisjak et al., 2016). It is also interesting to note that the UCNP@PMVEMA particles dissolved more in water compared to the neat particles (Figures 3 A, B). This phenomenon has been previously described for small 25-nm UCNPs and also for other polymer coatings probably due to their complexation with lanthanides (Plohl et al., 2017; Nahorniak et al., 2023). In contrast to water, dissolution of all particle types was significantly higher in PBS (Figure 3) because the reaction of phosphates with lanthanides accelerated the hydrolysis of surface atoms (Lisjak et al., 2016; Saleh et al., 2020). The F ¯ leakage from neat UCNPs reached 20 mol% after 44 and 7 h of incubation in PBS at 25°C and 37°C, respectively (Figures 3C, D). UCNP@Ale-PEG and UCNP@Ale-PDMA particles were more resistant to dissolution in PBS; at 25°C, *X*_F_ reached 20 mol% after 94 and 81 h, respectively (Figure 3C). In the case of UCNP@PMVEMA particles in PBS, *X*_F_ reached only 16 mol% even after 168 h. When the temperature was increased to 37°C, 20 mol% F ¯ was released from UCNP@Ale-PEG, UCNP@Ale-PDMA and UCNP@PMVEMA after 15, 14 and 43 h of incubation, respectively (Figure 3D). As a result, the dissolution rate of UCNP@PMVEMA in PBS at 37°C was lower (0.45 mol%/h) than that of UCNP@Ale-PEG (0.8 mol%/h), UCNP@Ale-PDMA (1 mol%/h) and neat UCNPs (1.3 mol%/h; Table 1). Similar results were also obtained at 25°C, but the dissolution rate of all particles was 2.5–6 times slower than that at 37°C due to the poor solubility of NaYF_4_. Thus, all protective coatings retarded the dissolution of UCNPs in PBS. Moreover, the determination of the molar fraction of released Y^3+^, Yb^3+^ and Er^3+^ ions (*X*_Y,Yb,Er_) supported the above statement that UCNP@PMVEMA particles were the most resistant to dissolution in PBS at 37°C. *X*_Y,Yb,Er_ of UCNP@PMVEMA, UCNP@Ale-PEG, UCNP@Ale-PDMA and UCNPs after 72 h of incubation was 1.46 ± 0.07, 1.57 ± 0.25, 2.46 ± 0.25 and 4.75 ± 0.63 mol%, respectively (Figure 4).

Furthermore, DMEM culture medium and ALF simulating the environment inside lysosomes with which the particles come into contact after endocytosis were used to model the dissolution of UCNPs under different *in vitro* conditions (pH, ionic strength and the presence of biomolecules). In DMEM, 1.9, 1.8, 1.8 and 3.3 mol% of F ¯ ions were released from UCNPs, UCNPs@Ale-PEG, UCNPs@Ale-PDMA, and UCNPs@PMVEMA particles, respectively, after 168 h of aging at 37°C (Supplementary Figure S7A). The nanoparticle dissolution in ALF was more pronounced than in DMEM, with *X*_F_ reaching ∼13, 8, 9 and 16 mol% for neat, Ale-PEG-coated, Ale-PDMA-coated and PMVEMA-coated UCNPs, respectively, after 168 h at 37°C (Supplementary Figure S7B). The slower dissolution in DMEM that in ALF was due to the formation of a protein corona on the particle surface, which inhibited their dissolution (Saleh et al., 2020). Thus, UCNPs can be expected to dissolve predominantly intracellularly and their toxicity will be related to the number of internalized nanoparticles and protective polymer coating.

When comparing the dissolution of particles in PBS, the UCNP@PMVEMA with the highest amount of coating were the most resistant to dissolution. However, in water these particles demonstrated the highest F ¯ release, suggesting that not only the amount of polymer but rather chemistry of coating and surface ζ-potential influenced the rate of particle dissolution. In contrast to PMVEMA with many carboxyl groups that can act as attachment sites of polymer with the particle, Ale-PEG and Ale-PDMA contained only one phosphonate group for binding with UCNPs. Therefore, a polymer with one anchoring group on the particle surface can more easily exchange with phosphate ions, which accelerates the hydrolysis of surface atoms and induces faster particles dissolution. This agreed with earlier report, where ligand containing four anchoring groups provided higher protection of UCNPs against dissolution than those with only two (Andresen et al., 2020). On the other hand, the negative ζ-potential of UCNP@PMVEMA particles can suppress the diffusion of phosphate ions to the nanoparticle surface due to Coulomb repulsions, inhibiting exchange reaction and particle dissolution. Moreover, the particle size seems to influence the rate of dissolution in aqueous media. In comparison to earlier report on the dissolution of 25-nm particles with the same coatings, the dissolution of hexagonal UCNPs in water and PBS at 37°C was lower (Nahorniak et al., 2023). In PBS, the dissolution of 120-nm particles was lower by ∼14 and 20%–24% for neat and all polymer-coated UCNPs, respectively. In water, the decrease in solubility was lower by 31, 37, 65% and 9% for UCNPs, UCNPs@Ale-PEG, UCNPs@Ale-PDMA and UCNPs@PMVEMA, respectively (Table 2). This was attributed to a smaller surface-to-volume ratio of 120-nm UCNPs, which agrees with the literature (Saleh et al., 2020). As a result, the dissolution of UCNPs is a complex phenomenon dependent on many parameters such as temperature, type of aqueous medium, nanoparticle size, surface chemistry and charge.

**TABLE 2:** Molar fraction of F ¯ ions (*X*_F_) dissolved in water or PBS at 37°C for 168 h and percentage of *X*_F_ from large (120 nm) versus small (25 nm) nanoparticles.

	*X*_F_ (mol%)				1-(*X*_F 120 nm_/*X*_F 25nm_) 100 (%)	
	Water		PBS			
Particles	25 nm	120 nm	25 nm	120 nm	Water	PBS
UCNPs	5.8	4.0	90.6	78.3	31.4	13.6
UCNP@Ale-PEG	3.3	2.1	73.1	55.9	36.9	23.5
UCNP@Ale-PDMA	6.2	2.2	73.8	56.0	65.1	24.1
UCNP@PMVEMA	4.9	4.5	48.4	38.4	8.7	20.7

*X*_F_ values for 25-nm UCNPs were taken from (Nahorniak et al., 2023).

### 3.5 Cytotoxicity of polymer-coated hexagonal UCNPs

To assess the suitability of UCNPs for biological applications, it is necessary to determine whether the individual particle types, specifically UCNPs, UCNP@Ale-PEG, UCNP@Ale-PDMA and UCNP@PMVEMA both without and with bound FITC are toxic to cells. Two cell types were used to assess cytotoxicity, rMSCs serving as a model of healthy non-tumorigenic cells and a C6 rat tumor cell line representing diseased tumorigenic cells. Cells in cell culture medium were incubated with particles at a concentration of 1–1,000 μg/mL for 24 and 72 h, and cell viability was examined using the Alamar Blue assay, one of the best *in vitro* assays for the determination of cell viability, cytotoxicity and cell proliferation. It is an alternative to tetrazolium dyes such as 3-(4,5-dimethylthiazol-2-yl)-2,5-diphenyltetrazolium bromide (MTT), and because it is non-toxic, it can also be used for long-term cell proliferation studies. Alamar Blue is based on the fluorometric redox indicator resazurin (7-hydroxy-3H-phenoxazin-3-one 10-oxide), a blue-colored non-fluorescent compound. After intracellular uptake, the oxidized resazurin is reduced to the fluorescent resorufin (7-hydroxy-3H-phenoxazin-3-one) due to the reducing environment of the cell cytosol. Resorufin produces bright red fluorescence with an excitation of 530–570 nm and emission of 580–610 nm; the fluorescence intensity is then used to quantify cell viability (Longhin et al., 2022).

**FIGURE 5:**
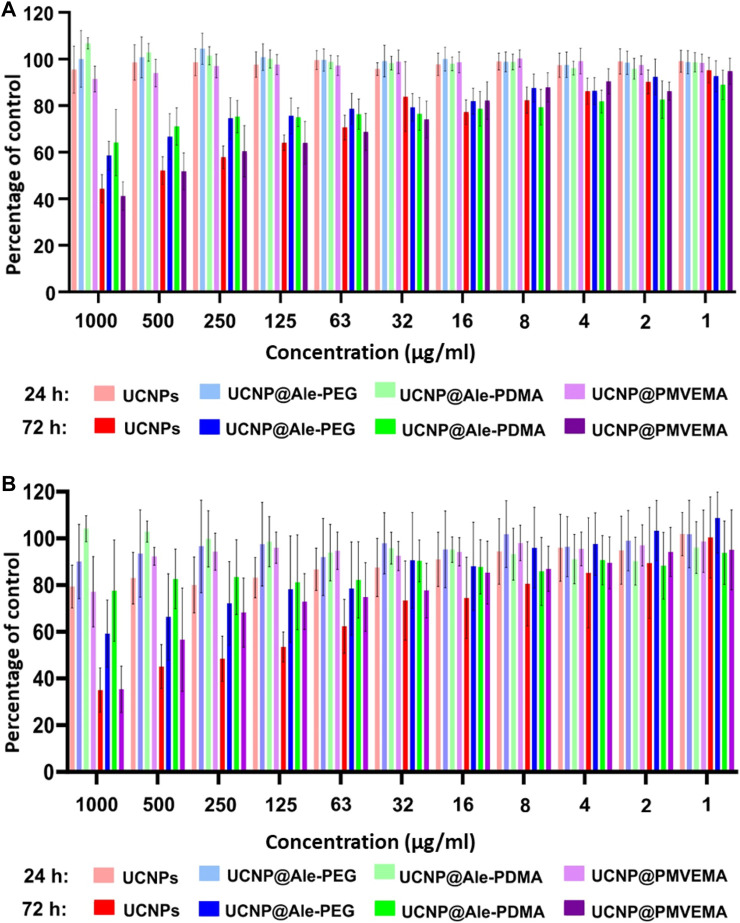
**(A)** C6 cell line and **(B)** rMSC viability in the presence of different concentrations of various UCNPs for 24 and 72 h.

The presence of nanoparticles in the medium for 24 h did not affect the proliferation of C6 cells regardless of the type of particle coating (Figure 5). Similarly, the viability rMSCs cultured for 24 h in the presence of different UCNPs at two highest concentrations (500 and 1,000 μg/mL) decreased only slightly (to 80%). Culturing C6 cells for 72 h in the presence of uncoated UCNPs and UCNP@PMVEMA particles at a concentration of 1,000 μg/mL resulted in a decrease in cell viability to 40%; with decreasing particle concentration, viability slowly increased to values reaching 80%–90%. Toxicity of UCNP@PMVEMA particles can be ascribed to their high solubility in water (Figures 3A, B) as well as in AFL. In contrast, cells cultured in the presence of UCNP@Ale-PDMA or UCNP@Ale-PEG nanoparticles at the two highest concentrations of 500 and 1,000 μg/mL maintained viability between 70% and 58%, while at lower concentrations cell viability was between 70% and 90%. Likewise, the viability of rMSCs cultured for 72 h in the presence of UCNPs and UCNP@PMVEMA particles was reduced to 30% and maintained <60% up to a concentration of 63 μg/mL. At lower concentrations of neat UCNPs and UCNP@PMVEMA, the viability ranged between 80%–90%. The rMSCs incubated with UCNP@Ale-PDMA maintained 80%–90% viability regardless of particle concentration. For the same cells incubated with UCNP@Ale-PEG at a concentration of 63–1,000 μg/mL, viability gradually decreased from 80% to 60%; at lower concentrations, rMSC viability was ∼90%. In addition, there was no difference in the viability of cells incubated with UCNPs either with or without FITC. These findings corresponded to the results of dissolution of UCNPs in ALF, as the best viability was achieved for particles coated with PEG and PDMA, which protected them well. In our previous study, a significant decrease in rMSCs and C6 cell viability (<20%; MTT assay) was observed already after 24 h of incubation with uncoated small spherical UCNPs at a concentration 1,000 μg/mL (Nahorniak et al., 2023). It should be mentioned that the toxicity of particles is generally influenced by a number of factors, including the ζ-potential, the particle size and the amount of lanthanide and fluoride ions released into the medium. A limitation of our study was that we did not have non-toxic polymer-coated particles with the same ζ-potential as the control. However, the ζ-potential of UCNP@Ale-PDMA particles was comparable to that of uncoated UCNPs, but the cytotoxicity of the former was significantly lower. Thus, it can be concluded that the cytotoxicity of UCNPs is not only determined by the ζ-potential but also by the dissolution of the particles.

### 3.6 Monitoring the internalization of polymer-coated hexagonal UCNPs by confocal microscopy

To demonstrate the concept of dual imaging, i.e., to visualize particle internalization in cells and particle distribution in the cytoplasm by confocal microscopy, a commonly used fluorescent dye, fluorescein isothiocyanate (FITC), was attached to UCNPs, which is useful for prospective *in vivo* animal control and histology. In addition, optical imaging allows for independent control, where nanoparticles can be excited throughout the organism to easily verify their biodistribution. The FITC was first modified with Ale to complex it with lanthanide ions on the particle surface. Its normalized photoluminescence spectrum and spectra of FITC-modified polymer-coated UCNPs under excitation and emission at 492 and 517 nm, respectively, were shown in Supplementary Figure S8. The main advantage of fluorescent particles is that they are visible in an epifluorescence microscope, which is part of the equipment of every culture laboratory, and therefore their uptake by cells can be monitored during cell culturing; it is also easier to detect particles in histological tissue sections. In addition, with excitation at 980 nm and emission at 542 and 656 nm, UCNPs enable deep tissue *in vivo* imaging.

**FIGURE 6:**
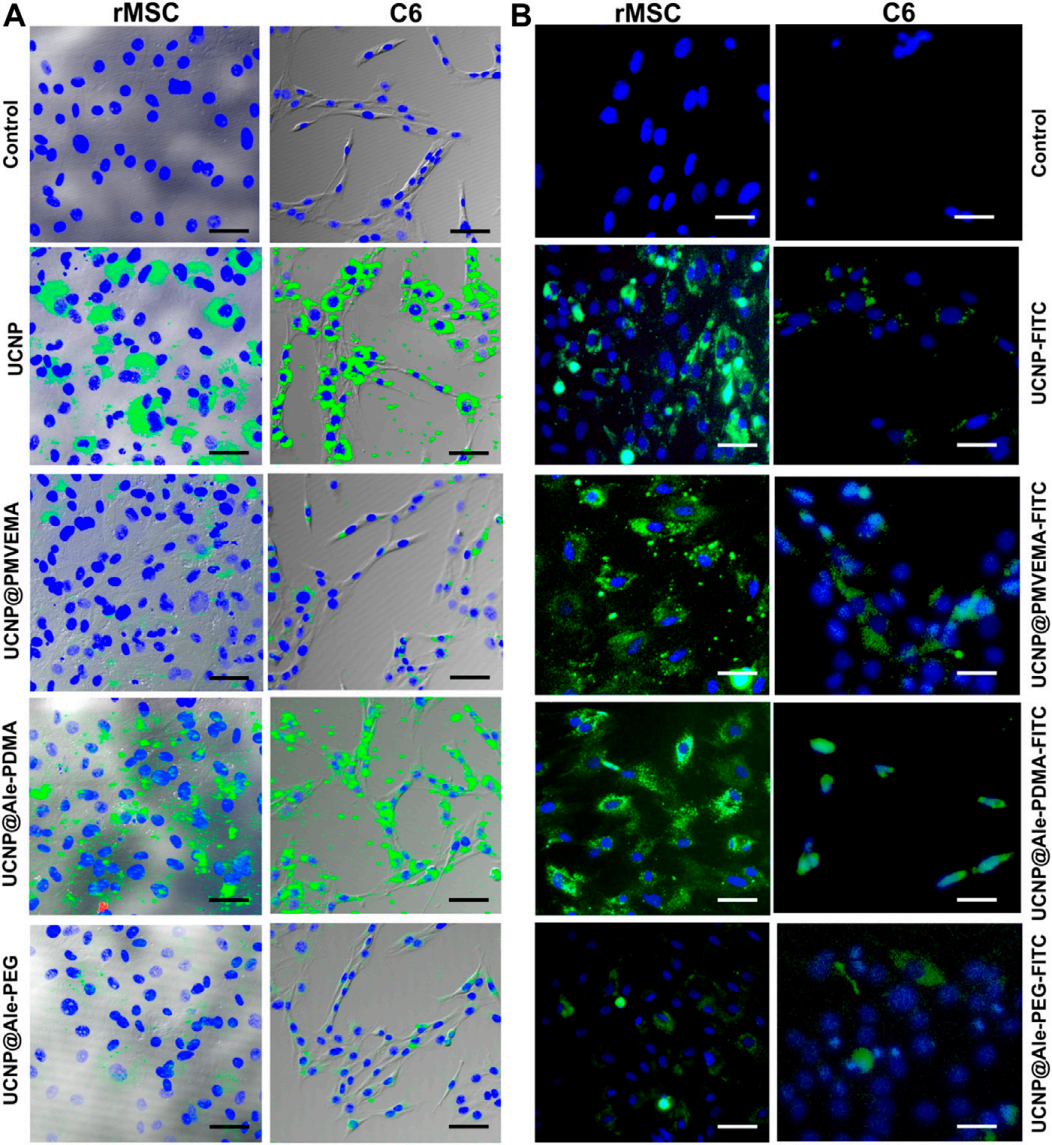
C6 and rMSC cells imaged by confocal microscope and overlaid with brightfield images **(A)** after 1 day of incubation with UCNPs, UCNP@PMVEMA, UCNP@Ale-PDMA, and UCNP@Ale-PEG particles (green). **(B)** FITC-modified UCNPs, UCNP@PMVEMA, UCNP@Ale-PDMA, and UCNP@Ale-PEG particles visualized in C6 and rMSC cells by fluorescence microscope after 24 h of incubation. The cell nuclei were stained with DAPI (blue). Cells not incubated with nanoparticles served as a control. Scale bar 50 µm.

**FIGURE 7:**
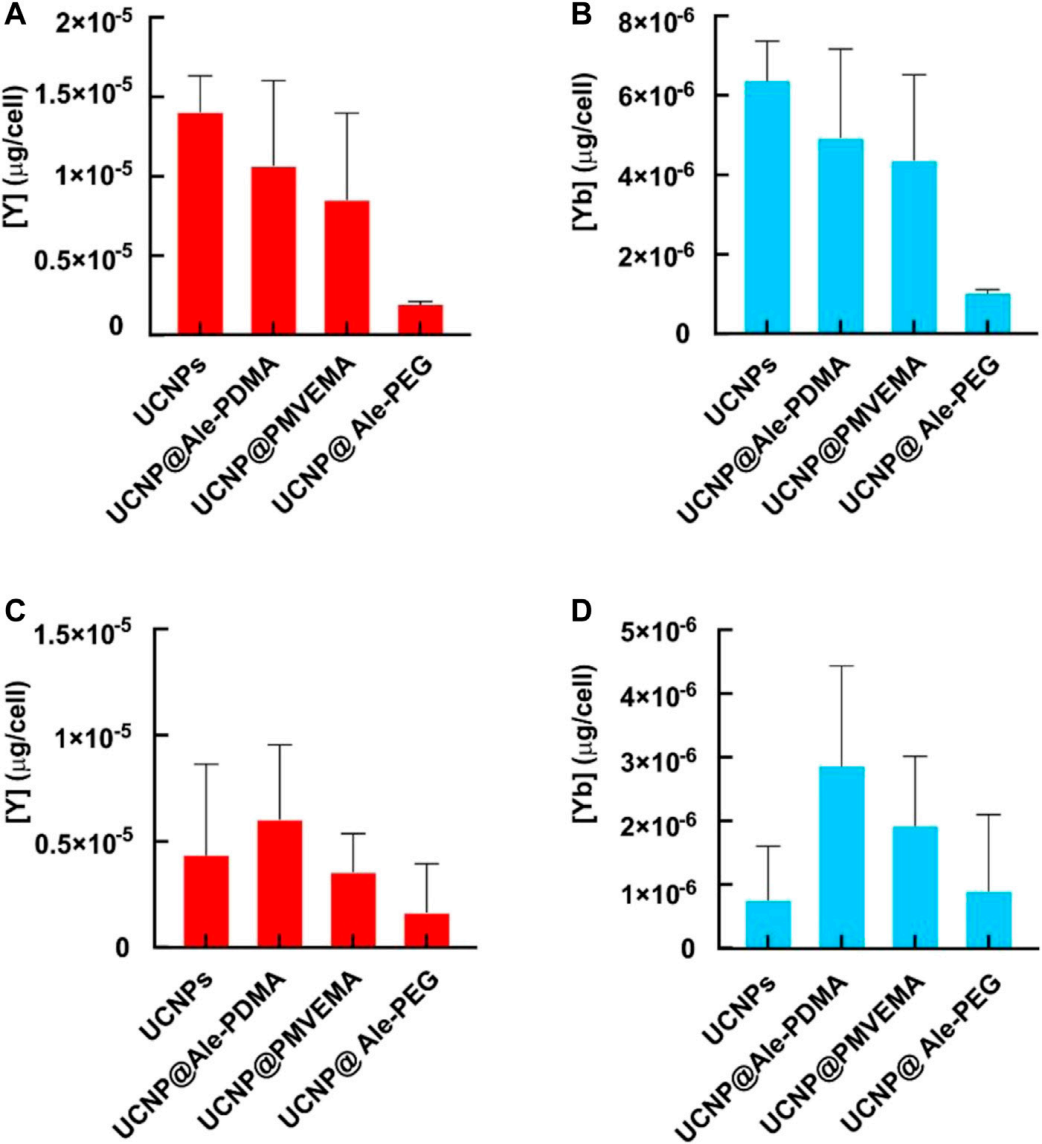
Amount of Y and Yb in **(A, B)** rMSCs and **(C, D)** C6 cell line after incubation with differently coated hexagonal UCNPs for 1 day according to ICP-MS.

To examine the uptake of all differently coated UCNPs, rMSC and C6 cells were cultured in the presence of particles at a concentration of 20 μg/mL for 24 h. Cells were then fixed with paraformaldehyde and imaged under confocal and epifluorescent microscope (Figure 6). Our results confirmed that particle uptake can be visualized by both confocal and fluorescence microscopy. The strongest signal in both confocal and fluorescence microscopy was observed in cells incubated with uncoated UCNPs. Of the coated particles, UCNP@Ale-PDMA were the most abundant in the cells, followed by UCNP@PMVEMA. UCNP@Ale-PEG particles were the least abundant in the cells because PEG is bioinert to a number of biological components found in the human body including proteins and does not exhibit cell-adhesive properties. There were generally more particles in the cytoplasm of rMCSs because they are usually larger than C6 cells. The signal was brightest in cells incubated with uncoated UCNPs, probably due to their high internalization (Figure 6). These findings are consistent with the results obtained by ICP-MS, which determined the concentration of Y and Yb in rMSCs and C6 cells after 24 h of culture with nanoparticles (Figure 7). The highest concentrations of Y and Yb were found in rMSCs containing uncoated UCNPs; Y and Yb concentrations ranged from 11.3 to 15.4 and from 5.2 to 7.0 pg per cell, respectively. The amount of Y per cell ranged from 4.9 to 15.5 pg and from 5.6 to 12.3 pg for UCNP@Ale-PDMA and UCNP@PMVEMA nanoparticles, respectively; similarly, the amount of Yb per cell was 2.6–7.0 pg and 2.8–5.9 pg for UCNP@Ale-PDMA and UCNP@PMVEMA nanoparticles, respectively. The only difference was found for UCNP@Ale-PEG particles, as PEG prevented their internalizing into cells and the concentrations of Y and Yb were in the range of 1.7–2.0 and 0.9–1.1 pg per cell, respectively.

In general, slightly lower concentrations of Y and Yb were found in C6 cells than in rMSCs (Figure 7). The highest amounts of Y and Yb were in C6 cells incubated with UCNP@Ale-PDMA nanoparticles for 24 h, 3.5–10.1 pg and 1.6–4.6 pg per cell, respectively. The average levels of Y and Yb in C6 cells labeled with uncoated particles were 4.4 and 0.7 pg/cell, respectively, while C6 cells incubated with UCNP@PMVEMA particles contained 3.6 and 1.9 pg of Y and Yb per cell, respectively. As in rMSCs, the lowest concentrations of Y (1.7 pg) and Yb (0.9 pg) were found in C6 cells containing UCNP@Ale-PEG nanoparticles. Comparison of the results of hexagonal UCNPs with previously published data on spherical nanoparticles coated with the same polymers (Nahorniak et al., 2023) shows that C6 cells cultured with hexagonal UCNPs contain orders of magnitude more Y and Yb than after culture with small spherical UCNPs. This difference was not observed for rMSCs, where Y and Yb concentrations were in a similar range, probably due to the larger size of rMSCs and their higher capacity for particle endocytosis. The size of the nanoparticles may also play a role; large particles contain higher amounts of Y and Yb, although their total number in the cell may not be greater than that of small spherical nanoparticles.

## 4 Conclusion

In this report, we present the design of uniform hexagonal NaYF_4_:Yb^3+^,Er^3+^ nanoparticles with a size of 120 nm that were coated with PEG-Ale, PDMA-Ale and PMVEMA polymers. The uniform size means that the particles have the same physicochemical properties, thus providing reproducible results in biomedical applications. After loading UCNPs with FITC, a two-color optical image with excitation at 494 or 980 nm can be obtained. This combination of two luminescence properties allows convenient and easy monitoring of biological processes *in vitro* and *in vivo*, such as particle uptake, tissue and tumor perfusion, or vascular leakage. Both colloidal stability and long-term dissolution of particles were systematically investigated in various aqueous media such as water, PBS, DMEM cell culture medium or ALF at 25°C and 37°C. Particle dissolution and colloidal stability were determined by potentiometry and DLS, respectively. To our knowledge, this is the first comprehensive study on the dissolution of UCNPs >100 nm in size. In contrast to small (25 nm) particles, the dissolution of large ones (120 nm) in water and PBS at 37°C was lower due to the smaller surface-to-volume ratio. PEG-Ale-and PDMA-Ale polymers provided a relatively good protection against particle dissolution in all tested media. While the UCNP@Ale-PEG particles were colloidally stable in water and DMEM, the UCNP@Ale-PDMA particles were stable only in water. Both particle types were unstable in PBS due to the exchange of polymer coatings with phosphates. In contrast, PMVEMA provided excellent colloidal stability of UCNPs in water, PBS and DMEM but suppressed their dissolution only in PBS, while accelerating dissolution in water, DMEM and ALF. Last but not least the cytotoxicity of particles was determined by Alamar Blue assay using C6 cells and rMSCs. Short-term (24 h) incubation of cells with UCNPs even at the highest concentration did not significantly affect cell viability. However, the viability of cells incubated with different types of particles for 72 h decreased to 40%–85% depending on the concentration. Neat UCNPs and UCNP@PMVEMA particles caused the greatest decrease. The highest particle uptake was seen with neat UCNPs, followed by UCNP@Ale-PDMA and UCNP@PMVEMA. Successful particle internalization and cell visualization was documented by green fluorescence of FITC-Ale-modified UCNPs inside the cells. Thus, it can be concluded that UCNP@Ale-PDMA and UCNP@Ale-PEG nanoparticles are the most promising in terms of further applications. Both particles were the least toxic but differed in their uptake into cells. While UCNP@Ale-PEG may find applications with prolonged circulation time when internalization into cells is not required, UCNP@Ale-PDMA is suitable for accumulation into, for example, tumor cells. In addition, surface-modified UCNPs can be prospectively loaded with various bioactive agents such as drugs or photosensitizers.

## Data Availability

The original contributions presented in the study are included in the article/Supplementary Material, further inquiries can be directed to the corresponding author.
