# Supplementary material for: Polymer-coated hexagonal upconverting nanoparticles: chemical stability and cytotoxicity
Source: Front Chem. 2023 Jun 23;11:1207984. doi: 10.3389/fchem.2023.1207984 (PMC10327433; doi:10.3389/fchem.2023.1207984)
Supplement: Supplementary file 1 [file DataSheet1.PDF]

Supplementary Material

Polymer-Coated Hexagonal Upconverting Nanoparticles: Chemical Stability and Cytotoxicity

Vitalii Patsula[1], Dana Mareková[2,3], Pavla Jendelová[2,3], Mykhailo Nahorniak[1], Oleksandr Shapoval[1], Petr Matouš[4], Viktoriia Oleksa[1], Rafał Konefał[1], Magda Vosmanská[5], Lucia Machová-Urdziková[2], Daniel Horák[1,*]

*** Correspondence:** Daniel Horák: horak@imc.cas.cz

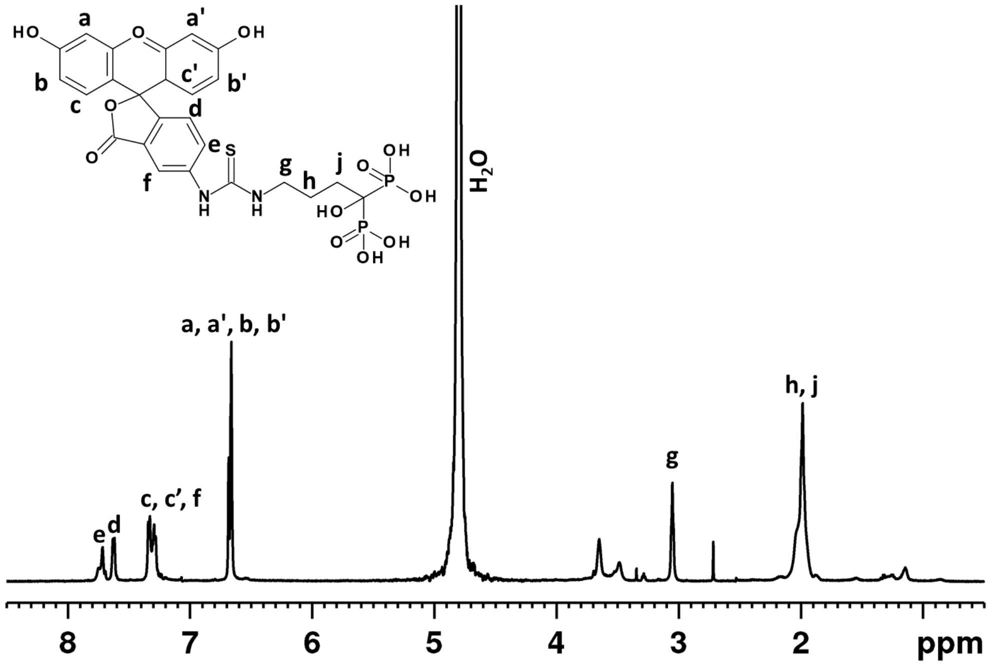

Figure S1. ^{1}H NMR spectrum of FITC-alendronate.

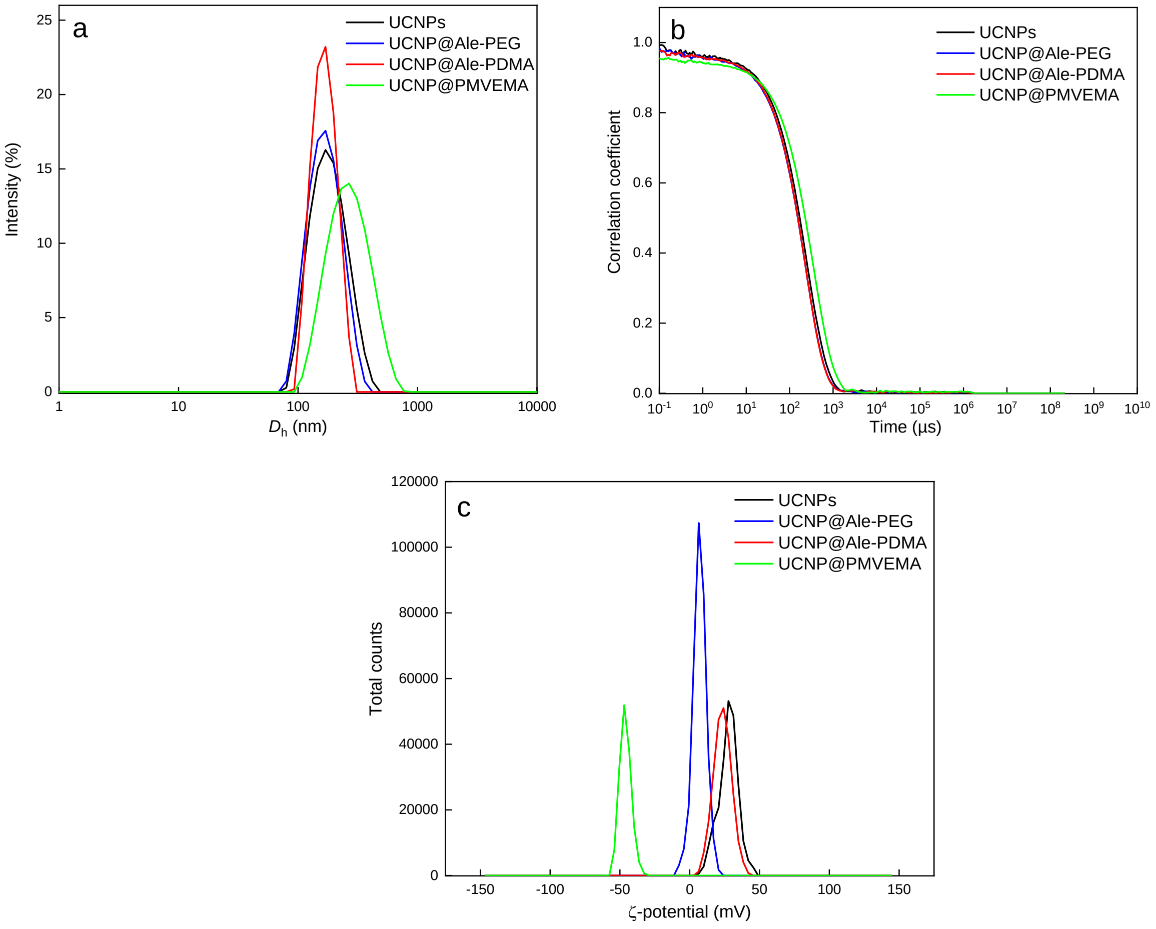

Figure S2. (a) Intensity-weighted particle size distributions with corresponding (b) correlograms and (c) ξ-potential distributions of various UCNPs in water (1 mg/ml).

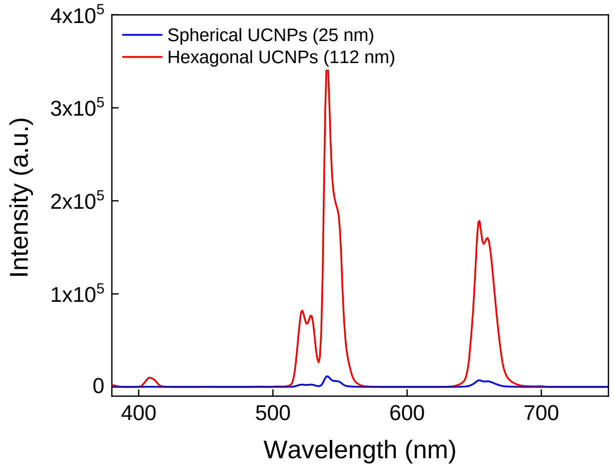

Figure S3. Upconversion photoluminescence emission spectra of uncoated spherical and hexagonal UCNPs (4 mg/ml of water) excited at 980 nm; 0.25 W/cm^2.

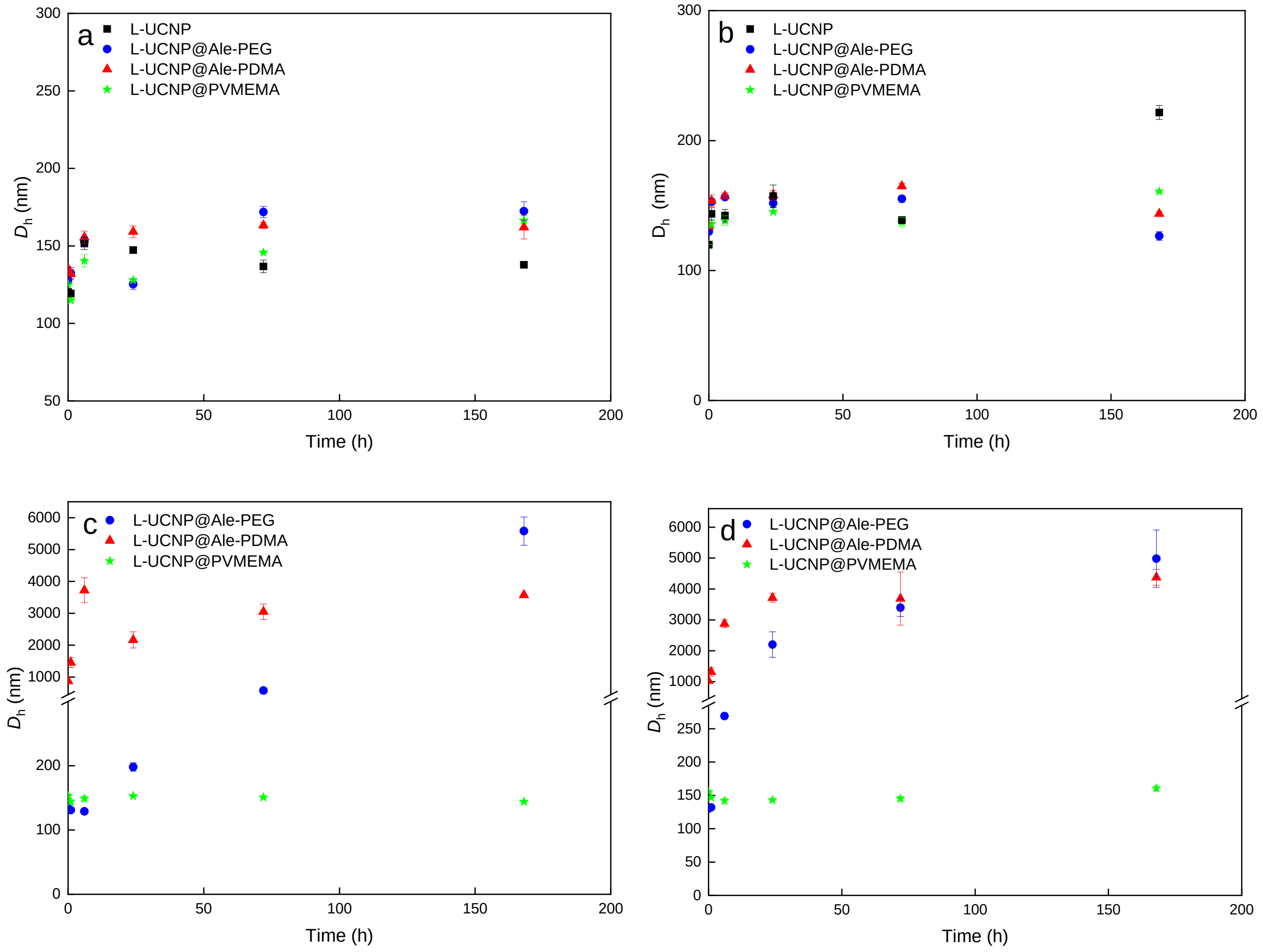

Figure S4. Time dependence of hydrodynamic diameter (D_h) of polymer-modified UCNPs in (a, b) water and (c, d) PBS (pH 7.4) at (a, c) 25 and (b, d) 37 °C.

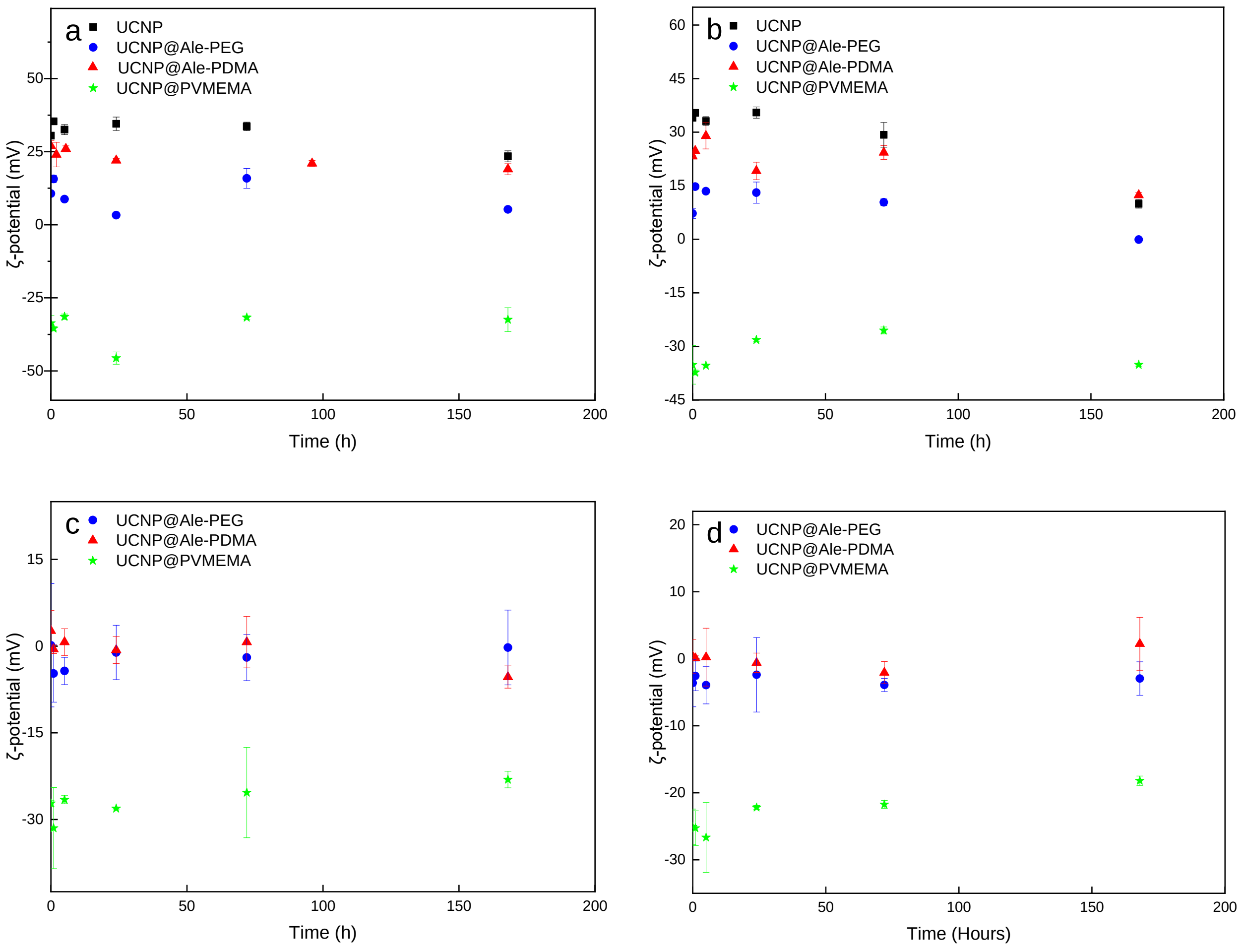

Figure S5. Time dependence of ζ-potential of differently coated UCNPs in (a, b) water and (c, d) PBS (pH 7.4) at (a, c) 25 and (b, d) 37 °C.

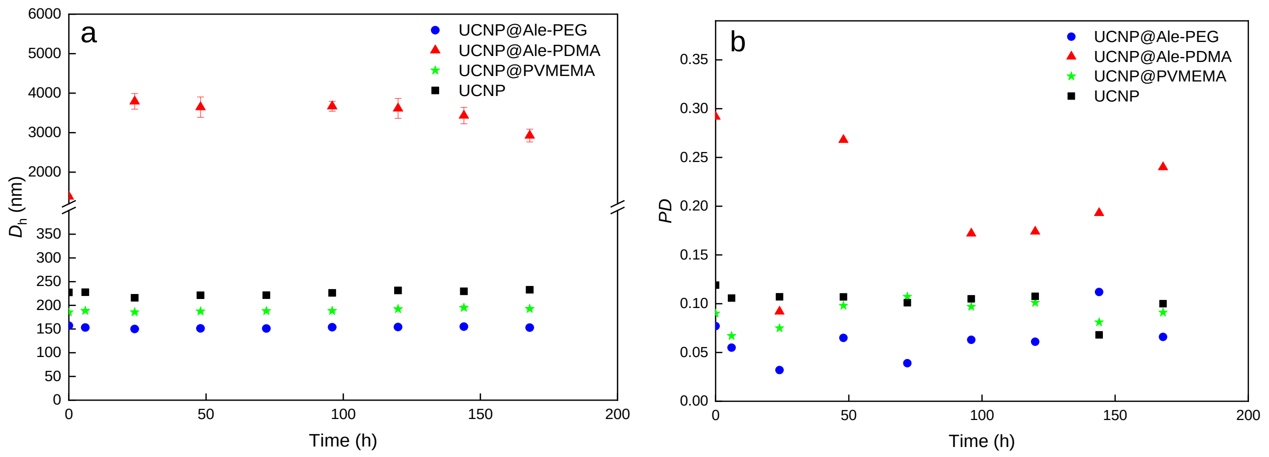

Figure S6. Time dependence of (a) hydrodynamic diameter (D_h) and (b) dispersity (*PD*) of neat and polymer-modified UCNPs in Dulbecco's modified Eagle's medium at 37 °C.

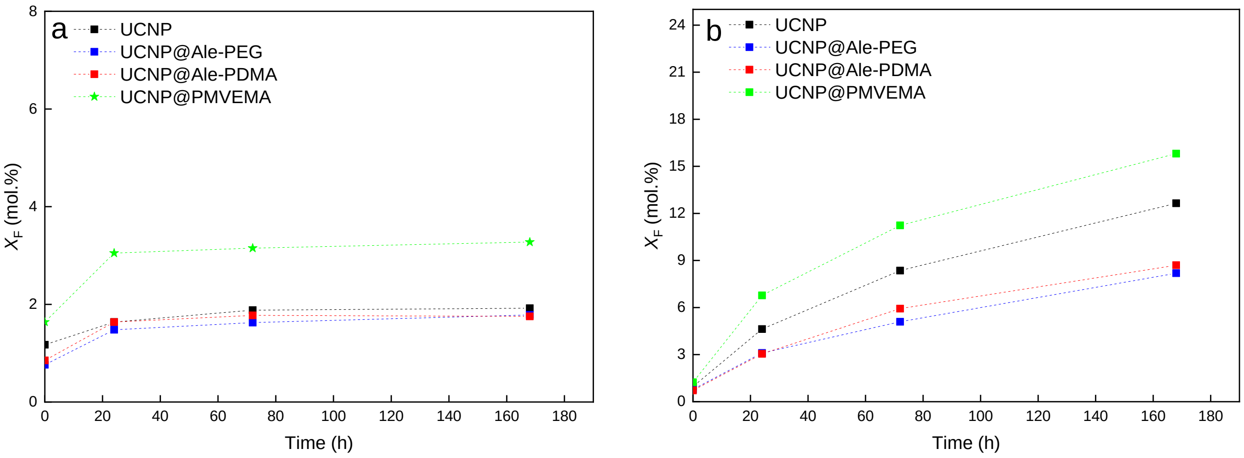

Figure S7. Time dependence of F¯ ion molar fraction (X_F) of differently coated UCNPs in (a) Dulbecco's modified Eagle's medium and (b) artificial lysosomal fluid at 37 °C. The standard errors of the means of X_F in DMEM ranged from 0.02 to 0.05 and in ALF from 0.02 to 0.04 mol.%.

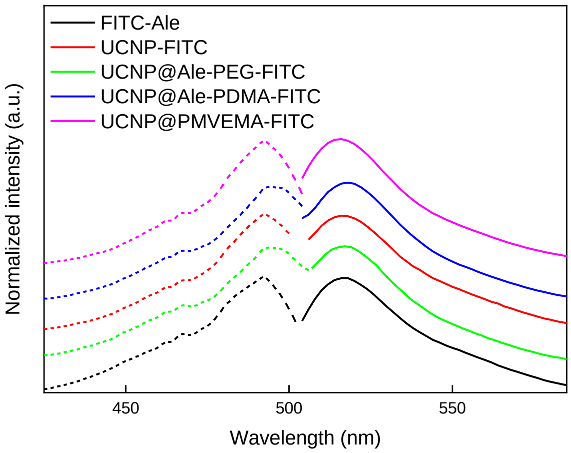

Figure S8. Normalized photoluminescence excitation (dashed curves; λ_{em} 517 nm) and emission (solid curves; λ_{ex} 492 nm) spectra of FITC-alendronate and FITC-modified neat and polymer-coated UCNPs.
